# What stops practitioners discussing medication breaks in children and adolescents with ADHD? Identifying barriers through theory-driven qualitative research

**DOI:** 10.1007/s12402-018-0258-9

**Published:** 2018-07-07

**Authors:** Kinda Ibrahim, Parastou Donyai

**Affiliations:** 1 NIHR CLAHRC Wessex, University Hospital Southampton NHS Foundation Trust, Faculty of Medicine, University of Southampton, Mailpoint 807, Tremona Road, Southampton, SO16 6YD UK; 2 Department of Pharmacy Practice, University of Reading, Reading, UK

**Keywords:** ADHD, Drug holiday, COM-B system, Decision aid, Barriers, Children and adolescent

## Abstract

National and international guidelines on the treatment of attention deficit hyperactivity disorder (ADHD) in children and adolescents call for annual reviews to assess continuing need for medication by considering brief periods without medication, referred to as ‘Drug holidays’. However, drug holidays are reactively initiated by families, or recommended by practitioners if growth has been suppressed by medication rather than proactively to check the need. There is little evidence of planned, practitioner-initiated drug holidays from methylphenidate. The aim of this study was to identify what stops practitioners from routinely discussing planned drug holidays from methylphenidate with children, adolescents, and their parents. Practitioners involved in shared-care prescribing for children and adolescents with ADHD in one UK County were included. Interviews with 8 general practitioners (GPs) and 8 Child and Adolescent Mental Health Service (CAMHS) practitioners were conducted. Transcripts were analysed qualitatively against the components of the Capability–Opportunity–Motivation–Behaviour (COM-B) model. Possible interventions for increasing prescribers’ engagement with planned drug holidays were considered in response. Multiple barriers to practitioner engagement in planned drug holidays from methylphenidate were identified. Capability, in terms of knowledge and skills, was not a barrier identified for CAMHS practitioners but was for GPs. Opportunity was a main barrier for both groups, who reported lack of time and the absence of educational material about drug holidays. Motivation was more complex to define, with CAMHS practitioners questioning the need for drug holidays and GPs being more accepting due to worries about long-term medication side effects as well as cost savings. ‘Education’ and ‘enablement’ interventions were identified as key activities targeting all three components, which could feasibly increase uptake of practitioner-initiated planned drug holidays from methylphenidate. The application of the COM-B system identified a number of key barriers to practitioner engagement with drug holidays in children and adolescents with ADHD. Accordingly, a number of interventions could be developed to facilitate change. For example, educating and training GPs about ADHD management and drug holidays, and developing a decision aid to help families make informed decisions about whether or not to implement drug holidays could be used.

**Electronic supplementary material:**

The online version of this article (10.1007/s12402-018-0258-9) contains supplementary material, which is available to authorized users.

## Background

Attention deficit hyperactivity disorder (ADHD) is characterised by hyperactivity/impulsivity and inattention, affecting around 5% of school-age children in the UK (NICE 2013). The diagnosis of ADHD is characterised by symptoms such as hyperactivity, inability to concentrate for a length of time, bad temper, problems completing tasks, organisation difficulties, and impulsivity (Kidd 2000). The condition is associated with many potential medical, emotional, behavioural, social, and academic consequences (NICE 2006). Most children are first diagnosed with ADHD when they enter school (Sax and Kautz 2003) with about three-quarters of those diagnosed being male (Schneider and Eisenberg 2006).

The treatment of ADHD involves multiple components including medical, behavioural, and educational interventions. Medication is the first-line treatment for school-aged children and young people with ADHD who have severe symptoms and impairment, and the second line for moderate impairment (NICE 2013). Worldwide, studies have shown that ADHD medications (both stimulants and non-stimulants) are effective in reducing ADHD symptoms and enhancing academic functioning in children receiving treatment (Hechtman et al. 2004; Pietrzak et al. 2006; Wilson et al. 2006). However, there has been a debate about the long-term side effects versus advantages on child’s schoolwork and achievement with ADHD medications (Sharpe 2014). An 8-year prospective follow-up of children treated for ADHD in a multisite study (MTA—Multimodal Treatment of Attention Deficit Hyperactivity Disorder) showed no long-term advantage of medication beyond 2 years in most cases (Molina et al. 2009). Plus, the short-term adverse effects such as suppression of appetite and growth in children and problems with sleeping present legitimate concerns for some families (Zachor et al. 2006; Faraone et al. 2008).

Whereas some parents tend to continue giving their children medication on a long-term basis, in a process described as ‘doing what helps most’ (Cormier 2012), others allow breaks from medication on some days (mostly non-school days), known as ‘drug holidays’ (Wilens et al. 2005; Hugtenburg et al. 2005; Dosreis et al. 2003). Drug holidays are ‘deliberate interruption of pharmacotherapy for a defined period of time and for a specific clinical purpose’; in ADHD practised mostly on non-school days such as weekend and/or school holidays (Howland 2009). When a drug holiday is being considered in ADHD, it typically concerns treatment with stimulant medications not atomoxetine or the alpha 2-agonists (Weed 2016). A qualitative study identified two types of practices: families who follow the ‘school-time medication pattern’ where they medicate their children only during the school week; and those who follow ‘all-time medication pattern’ where they give their children the medication every day, all the time (Kinda et al. 2016). There are seasonal patterns associated with using stimulant medication that coincide with school holidays (Shyu et al. 2016; Cascade et al. 2008). A systematic literature review found that between 25 and 70% of families follow the school-time medication pattern (Ibrahim and Donyai 2015). Different factors have been reported to affect the pattern adopted by families such as parents’ ability to cope with the child’s behaviour, the child’s academic progress, the location of the child’s problematic behaviour, severity of symptoms, and parental beliefs about the medication (Kinda et al. 2016).

The systematic literature review identified four benefits from practising drug holidays from ADHD medication including: assessing the need for medication, managing and preventing medication side effects, managing drug tolerance (the need to increase the dose with time to obtain the same therapeutic effect), and negotiating medication continuation or discontinuation (Ibrahim and Donyai 2015). The review suggested that doctors should discuss planned drug holidays with some if not all families at some point in the treatment of the child as a positive approach. Longer breaks from ADHD medication were reported to enable child growth, while shorter breaks could reduce insomnia and improve appetite. The Institute of Clinical Systems Improvement (ICSI) recommends that linear growth impairment as a result of ADHD treatment might be managed by limiting stimulant to high-priority needs, for example by trying weekend or vacation ‘drug holidays’ (ICSI 2010). Similarly, the European clinical guidelines for hyperkinetic disorder state, ‘If there are indications of growth retardation, drug holidays (e.g. during the summer vacation) are recommended’ (Taylor et al. 2004).

In addition, the National Institute for Health and Care Excellence (NICE) guidelines in the UK and guidance by the American Academy of Child and Adolescent Psychiatry (AACAP) in the USA make reference to intentional drug holidays in ADHD (NICE 2013; AACAP 2007). The AACAP guidelines suggest that clinicians should discuss the continuing need for medication with families if the patient with ADHD has been free of symptoms for at least 1 year. However, NICE recommendations about planned breaks from medication in children with ADHD and their families are less direct, stating ‘effect of missed doses, planned dose reductions and brief periods of no treatment should be taken into account and the preferred pattern of use should also be reviewed’. Nationally, and certainly within the setting of this study, methylphenidate shared-care arrangements ask both CAMHS doctors and general practitioners to discuss a 2-week drug holiday after 2 years of stimulant use to test the continuing need for treatment. This protocol is necessary for CAMHS to get the payment from the Commissioning for Quality and Innovation (CQUIN) in primary care. The system was introduced in 2009 to make a proportion of health care providers’ income conditional on demonstrating improvements in quality and innovation in specified areas of patient care. Shared-care is a particular arrangement which allows the transfer of clinical responsibility from a specialist service (e.g. CAMHS) to general practice, where prescribing by the GP is supported by a shared-care arrangement (NHS 2018).

Despite the national and international recommendations and the evidence of possible benefits from planned drug holidays, most planned drug holidays seem to be initiated by parents (Kinda et al. 2016; Ibrahim and Donyai 2015; Brinkman et al. 2009). Only 30 and 60% of practitioners in the USA and the UK, respectively, would consider discussing breaks from medication annually with families of children with ADHD (Salmon and Kemp 2002; Stockl et al. 2002). Therefore, the purpose of this study was to explore and identify the barriers for practitioners in a UK setting to discuss and undertake formal planned drug holidays from ADHD medication with a focus on testing the continuing need for medication. Identifying these barriers could possibly inform the selection of suitable interventions to increase the implementation of planned drug holidays under the supervision of health expertise.

## Methods

### Design

This was a qualitative research study utilising individual interviews. Transcripts were analysed thematically (Sadler et al. 2010) against the components of the Capability–Opportunity–Motivation–Behaviour (COM-B) model (Michie et al. 2011), to identify barriers to the uptake of planned drug holidays by practitioners. This approach was taken because it can provide a systematic way to move from behavioural analysis to identifying potential interventions which might bring about change. The COM-B system explains that human behaviour is best understood through the interaction between three main components: capability (physical and psychological), opportunity (social and physical), and motivation (reflective and automatic) (Michie et al. 2011) (see Table 1).

**Table 1:** Description and definition of the COM-B components

COM-B components		Definition
Capability	Physical psychological	Capability refers to an individual’s psychological and physical ability to get involved in the specific activity under concern and it includes having the knowledge and skills necessary to engage in the activity
Opportunity	Physical social	Opportunity is the different external factors that make the behaviour possible or prompt it. Opportunity barriers are external constraints on a provider’s enactment of a behaviour
Motivation	Reflective automatic	Motivation is defined as the brain processes that boost and direct behaviour including: habitual processes, emotional response, as well as analytical decision making

The Chair of the South Central, Berkshire NHS Research Ethics Committee (REC) advised that the research did not require NHS REC review because it involved interviews with NHS staff and not patients. The study was reviewed and approved by the University of Reading REC (reference UREC 12/18), the Berkshire NHS Research & Development office (letter of access granted 22/06/2012) and the Thames Valley Primary Care Research Partnership (reference TV84).

### Sample

Sixteen health care practitioners were purposefully sampled and contacted. The goal of purposeful sampling as described by Bryman (2012) is ‘to sample cases/participants in a strategic way’. Prescribing stimulants (e.g. methylphenidate) and other ADHD medications for children and adolescents is governed by shared-care arrangements between primary- and secondary-care professionals in the locality of this research (a UK county), where an annual discussion of a 2-week drug holiday is recommended. Therefore, the sample compromised of Child and Adolescent Mental Health Services (CAMHS) practitioners and general practitioners (GPs) in the study setting who responded to the invitation letter. Participants were recruited until no more new and significant concepts emerged (i.e. sampling saturation) (Fusch and Ness 2015). Data saturation was achieved by constantly comparing transcripts and checking whether any new data or concepts emerged. According to the literature in qualitative research, a total of 16 participants were considered sufficient (Boddy 2016). CAMHS practitioners were approached via a mutual resource (a CAMHS specialist consultant) who emailed an invitation letter and information sheet to all practitioners within a community and mental health hospital trust. Eight CAMHS practitioners (five female, three male) from four NHS CAMHS clinics across four towns took part in the study. These practitioners see similar patients and receive referrals according to their locality. GPs were recruited via an invitation letter and information sheet posted to 150 GPs selected at random from publicly available NHS addresses in the same county. Six GPs agreed to take part in the study. The ‘snowball’ technique was also used to recruit a further two GPs (Sadler et al. 2010) via the already-recruited GP participants. Eight GPs (two female, six male) from six different practices across four towns took part in the study.

### Procedure

Semi-structured interviews were conducted with the 16 practitioners using an interview schedule focusing on exploring beliefs about medication and personal experiences with ADHD drug holidays (see additional file 1). The semi-structured interview guide was drafted and refined by agreement with an external CAMHS consultant before being piloted with three volunteer participants to ensure workability and feasibility of the questions. The interview guide used open ended questions allowing flexibility to probe and ask questions in response to the conversation. Written consent was obtained from each participant before the interview, which was always held in a private office in the participant’s workplace. Interviews averaged approximately 20 min (ranged 10–30 min) in duration with GPs and 40 min (range 30–47 min) with CAMHS practitioners.

## Analysis

All interviews were audio-recorded, transcribed verbatim, and then anonymised. Data were analysed independently by the authors using the COM-B model as a basis following the steps of thematic analysis (Braun and Clarke 2006). Analysis involved a number of phases: familiarisation with the data, coding, searching for themes, reviewing themes, defining and naming themes, and writing up. After familiarising with data, the authors independently coded the data from each transcript and assigned initial ‘code names’. The researchers then compared their coding and resolved discrepancies by discussion. After agreement had been reached, the identified and named codes were allocated to match the COM-B components. This required the researchers to re-read data within the codes and then allocate the codes to the appropriate components. Data were imported into NVivo 10 to facilitate data analysis and code grouping.

The trustworthiness of the findings was established by ‘member checking’. First, each participant was sent the transcript of their own interview to ensure its accuracy, and very minimal revisions were needed. Secondly, a detailed report of the main findings was sent out to all participants asking for their feedback. Six responses were received, and participants appeared to agree with the main findings of the study. In addition, the study findings were presented to a panel of 12 CAMHS practitioners in the study county in one of their monthly meetings. There was a general agreement about the validity of the results in explaining the real practice of medication use and the exercise of planned drug holidays from ADHD medication among children and adolescents.

## Results

The analysis of in-depth interviews using the COM-B system unveiled a diverse range of practitioner experiences with ADHD medications and the barriers to discussing/initiating drug holidays in children and adolescents with ADHD to test the continued need for medication (see Table 2).

**Table 2:** Mapping of behaviour change barriers for practitioner-initiated drug holidays to theoretical constructs

COM-B system components	Barriers for CAMHS to discuss and undertake drug holidays	Barriers for GPs to discuss and undertake drug holidays
Physical capability	N/A	**Lack of skills and training about ADHD and drug holidays** We learnt about this when we were at medical school, and pick up bits and bobs from reading, but real knowledge No, we haven’t, no. (GP 1) I suspect most GPs are not particularly skills at ADHD. I don’t feel skilled at it (GP6) ‘we ought to have training sessions on, we’re talking about holidays and when to stop and when, so we can actually justify with evidence what we’re doing (GP 2)’
Psychological capability	N/A	**Unfamiliarity with guidelines’ and difficulties accessing them** No, I think perhaps that it would be useful for GPs to be more aware of the guidelines or to have the right guidelines more easily available. Well, they probably are but it’s just knowing where to find them because we get sent so many bits of information that it, it’s a constant thing (GP5)
Social opportunity	**Parents’ rejection to consider breaks from medication due to concerns about interrupting the treatment** Most of the parents don’t want to stop medication…Do you want to try them, the treatment withdrawal trial, but I think it’s still parents views that it’s not helping (CAMHS 7)	N/A
Physical opportunity	**Lack of educational materials about drug holidays** I don’t have any material available but I tell them to try, start trying this the weekend first, then try it more for, say 1 week or two, or if everything is ok then try it more and see how it goes (CAMHS 4)	**Time constraint** You could always make a case, I know, saying, oh, we should be involved more, but I think the truth is that it’s difficult because of the time constraint (GP 5) I mean basically my role is to check, is everything OK? Are you still taking the tablets? Are there issues with compliance? I’ll have a 10 min, per patient (GP1)
Reflective motivation	**Perceiving their role as to make enquiry about drug holidays and empower families to make their own decision** I, well I say, I mean my whole line is that I’m not there to say you have to do this or you don’t have to do this, I’m there to advise, give information, support where possible. If a parent says, no way am I going to have a break, then that is their decision (CAMHS 1) **Uncertainty about the benefits of drug holidays** So I don’t think a withdrawal always is helpful. In many cases the withdrawal is not helpful, yeah, so we have to be careful (CAMHS 4) I think that two-thirds of the children on medication then end up going back on medication (CAMHS 5) **Disagreement with the content of the guidelines regarding and perceiving drug holiday more advisable at older age** Well yeah, I think, well I think, yeah, it’s very rarely considered for anybody young, it’s more, I think, useful in teenagers…So I think it’s very difficult for a young child to make sense of withdrawing them, I think it’s the parents’ decision (CAMHS 2) **Viewing ADHD as a chronic disease that need constant treatment** It’s an interesting one isn’t it? Because if you’ve been diagnosed with diabetes you don’t tend to take 2 weeks off your insulin and I just sort of, it’s interesting so I wonder where that idea has come from about why we might withdraw medication (CAMHS 7)	**Perceiving their role as limited to referral and continue prescribing** My role I see as signposting, I’m not trained in child psychiatry (GP2) I think, it’s not something that I would initiate, of course, but my role is to repeat prescribing (GP 6) **Relying heavily on specialists** We do what we are told, do you know what I mean? It’s, because it’s such a specialised thing and I think, I didn’t receive that much training in it either (GP 8) No, I’m all, I’m waiting for the initiative to come from secondary care (GP1) **Concerns about abrupt withdrawal symptoms** I think the withdrawal has to be done slowly, just like an antidepressant, with close supervision (GP 8)
Automatic motivation	**Planned and unplanned drug holidays from ADHD medication are initiated by many parents, which makes practitioner-initiated drug holidays unnecessary.** Now any person who’s worked with ADHD will tell you the parent will occasionally forget to take the medication, give this child medication, and they will see the outcome, so they will notice, or the teacher will know that this child has not been medicated today. So most often you don’t need to do this specific 2 weeks (CAMHS 2) Quite often parents have tried it anyway, they said, well we just wanted to see what would happen (CAMHS 8) **Feeling that most children with ADHD need medication and are unable to function without it.** I don’t remember more than two or three cases who actually stopped and completely went out of without complaining about this (CAMHS 4)	N/A

### Identifying barriers to practitioner-initiated planned drug holidays

1. Capability

Practitioners’ self-assessment of their capability to discuss and initiate a break without ADHD medication in children and adolescents varied according to specialty. CAMHS practitioners had the necessary knowledge and skills to discuss and implement formal drug holidays with families. They were aware that they need to have formal discussions on an annual basis to check the continued need for medication, and that this was necessary for them to receive payment from the Commissioning for Quality and Innovation (CQUIN) in the Primary Care Trust. However, this did not appear to impact on their actual practice of initiating drug holidays themselves. Two CAMHS practitioners described that unintentional break from medication reported by parents was documented by CAMHS practitioners as effective trials without medication when filling the CQUIN form as shown below:

So quite often I will find that I’m recording in the file that there was an effective trial off medication even though it wasn’t planned (CAMHS 8)

So it’s unusual situation where people have been asked to do this but certainly in my mind, when we did the CQUIN for a whole year and nobody wanted to stop the medication (CAMHS 2).

On the other hand, most GPs were unfamiliar with the specific recommendations about planned drug holidays. Lack of awareness of guidelines was assigned to different reasons such as: difficulties accessing guidelines and shared-care protocols, and general work pressure and document overload. GPs thought they lacked skills in relation to ADHD management and were not prepared to initiate a break from medication without specialist involvement, unless they received specific training as expressed by the following GP:

We ought to have training sessions on, we’re talking about holidays and when to stop and when, so we can actually justify with evidence what we’re doing (GP 2).

2. Opportunity

Physical opportunity and social opportunity were identified as important barriers for both GPs and CAMHS practitioners to implement and adopt the recommendations about planned drug holidays. Time constraint was a major barrier for GPs to consider planned drug holidays but it was not an issue for CAMHS practitioners. Regular reviews (every 6 months) and long appointment slots allowed CAMHS practitioners to carry out a full detailed review and gave them the opportunity in theory to discuss planned drug holidays with families. In contrast, GPs reported that 10-min appointments were only enough to repeat the prescription but not to discuss in detail the idea of trying to withdraw medication. The following quote clarifies this barrier:

You could always make a case, I know, saying, oh, we should be involved more, but I think the truth is that it’s difficult because of the time constraint (GP 5).

When CAMHS practitioners discuss the idea of considering a break from ADHD medication, they do it verbally without the use of any other aids. There was no consensus guideline in relation to the information provided to families about ADHD drug holidays and so the discussions are left to the individual practitioner as illustrated in the following comment:

I don’t have any material available but I tell them to try, start trying this the weekend first, then try it more for, say one week or two, or if everything is ok then try it more and see how it goes (CAMHS 4)

Thus, the perspective of individual CAMHS practitioners and their perceptions towards drug holidays could possibly impact on the information provided to families and their acceptance. The decision of whether or not to take a planned break from medication is made by parents of children with ADHD, specifically with younger patients. CAMHS practitioners perceived their role was to discuss trying to stop the medication temporarily and to empower families to make the decision themselves. Negative parental perceptions towards drug holidays did not allow some CAMHS practitioners to further explore a planned drug holiday and others initiate weekend drug holidays without the practitioner’s recommendation as demonstrated in the following comments:

Most of the parents don’t want to stop medication…Do you want to try them, the treatment withdrawal trial, but I think it’s still parents views that it’s not helping (CAMHS 4)

CAMHS practitioners reported some reasons for parental rejection of planned drug holidays including worries about the child’s academic life, negative experiences with unintentional non-adherence accidents, fears of teachers’ complaints, parents’ inability (or fears of inability) to cope with their children without the medication, and worries of going back to the previous state before starting the medication. The following comment from one CAMHS practitioner reflected this clearly:

Other parents just think, well no I’m going to go back to doing, they’re going to go back to being what they were before, without even trying to have a holiday break (CAMHS 6)

3. Motivation

Despite unawareness of specific recommendations about planned drug holidays, all GPs showed positive opinions towards the concept for a variety of reasons. Concerns about stimulants’ unknown long-term effects, especially on a child’s brain, made four GPs favour the idea of stopping the medication temporarily.

Well do they still need it? Why should I give medication which is unnecessary? Should we be doing that especially in children with growing brains and all this sort of thing? (GP 1)

In principle I think it’s a good idea, yes. You, ultimately you would like all children with ADHD to be off medication if necessary, either because their condition improves or medication is no longer needed (GP 3)

I think one needs to be aware of the side effects and, as I say, from my perspective, I’m always keen to try and withdraw any medication with children, particularly a medication like methylphenidate if one worries about what the effects might be (GP 5)

Planned drug holidays were seen by five GPs as an opportunity to identify children who might not need to take the medication anymore and avoid putting them on unnecessary medication which could possibly save NHS resources as reported by the following GP:

Well as I said, for me the instance is a psycho-stimulant that works on the central nervous system, secondly the liver accumulates all the chemicals we put into it. Thirdly why prescribe when you don’t have to in terms of cost on NHS? (GP 2)

However, CAMHS practitioners showed varied perceptions. Five CAMHS practitioners had positive attitudes towards drug holidays and thought it was good practice to test the continuing need for medication. One CAMHS doctor argued that the need for medication can’t be assessed unless medication is stopped ‘*if you don’t stop you don’t know*’, while three CAMHS practitioners questioned the need for implementing drug holidays and were less motivated to actually exercise it. One viewed ADHD as a chronic disease similar to diabetes and hypertension that needs to be treated continuously. Others believed that the effectiveness of medication is well checked by parents either by intentionally taking a break from medication or unintentionally when forgetting to give the medication:

It’s an interesting one isn’t it? Because if you’ve been diagnosed with diabetes you don’t tend to take two weeks off your insulin and I just sort of, it’s interesting so I wonder where that idea has come from about why we might withdraw medication (CAMHS 7)

They (parents) are good checking it out themselves, they don’t need us to tell them when to try it. I think it’s, that’s my perception of why it’s happened (CAMHS 2)

Some CAMHS practitioners felt that a drug holiday is generally unhelpful and that most children with ADHD can’t function well without the medication. They expressed worries about the possible consequences of stopping the medication even temporarily on family life and the child’s academic achievements. Some disagreed with the guidelines requirements and argued that planned drug holidays were not preferable at younger age and more advisable among adolescents. They believed that these breaks could allow self-assessment of ability to manage without the medication as demonstrated below:

Somebody’s ten year old, and he started medication at eight year old, then obviously the psychological intervention they may not be able to use it correctly. I don’t know but comparatively, compared to 10 years stopping, compared to 14 years stopping, the medication is more needed (CAMHS 4)

The uptake of planned drug holidays from ADHD medication tied in with beliefs about perceived roles. GPs’ viewed ADHD a specialist area and perceived their role to be limited to initial referral of families for CAMHS assessment and later to continue/repeat prescription. They had concerns about the possibility that stopping stimulant medication abruptly could be associated with withdrawal effects which made them reluctant to initiate drug holidays themselves. They believed that ADHD medication (specifically stimulants) acts similar to antidepressants by interfering with dopamine and noradrenaline levels in the brain and could possibly have similar adverse events. They showed preference that the initiative should come from the specialist and withdrawal needed to occur over slowly a prolonged period. However, abrupt withdrawal of ADHD medication was not a concern for CAMHS practitioners who believed that the medication is either in or out of the body system:

I would be reluctant to withdraw it without advice from the consultant. Yes, if you withdraw a stimulant abruptly then clearly there could be withdrawal effects from it and you would want to manage those (GP 3)

## Discussion

Using the COM-B model allowed identification and understanding of the barriers that impact on the practice of planned practitioner-initiated drug holidays. CAMHS practitioners appeared to have the capability to initiate planned drug holidays, whereas lack of knowledge and skills about ADHD in general and specifically about drug holidays was a vital barrier for GPs. Opportunity was a main barrier for both groups. Time constraint, parental disinclination to stop medicating their children, and lack of educational/information resources about ADHD drug holidays were all reported as potential barriers. Motivation was more complex to define for both CAMHS practitioners and GPs. CAMHS practitioners were less motivated to initiate planned drug holidays and some felt families should be empowered to make such a decision. However, GPs were more motivated due to worries about long-term medication side effects as well as cost savings but felt uncomfortable to withdraw the medication themselves.

GPs lacked motivation and capability to initiate themselves a break from medication due to lack of knowledge and training about ADHD in general and drug holidays in specific. Several studies worldwide have reported inadequate knowledge of GPs about ADHD and its treatment (Ghanizadeh and Zarei 2010; Jawaid et al. 2008; Lian et al. 2003; Louw et al. 2009; Shaw et al. 2003; Salt et al. 2005). GPs elsewhere have also expressed low levels of interest in becoming highly involved in ADHD care (Shaw et al. 2003; Salt et al. 2005). Thus, ‘education’ and ‘enablement’ interventions designed to improve the knowledge and skills of GPs about ADHD, its management, and how to withdraw the medication could increase their confidence in their abilities to manage children with ADHD and could motivate them to engage more in discussions about drug holidays with families of children with ADHD. Educational outreach visits, whereby trainers visit clinicians where they practise and provide them with information to change how they practise, are reported to improve the delivered care for patients (O’Brien et al. 2007). A Cochrane review found that interactive training results in moderately large changes in professional practice (Forsetlund et al. 2009).

Incentivisation also could be a possible intervention to target lack of GPs’ involvement. Some of the GPs interviewed in this study reported that he would consider discussing and undertaking planned drug holiday from ADHD medication if they were to receive incentives. However, as shown earlier, incentivisation interventions (the Commissioning for Quality and Innovation CQUIN incentives) were not thought by CAMHS practitioners to increase the uptake of practitioner-initiated drug holidays. Setting up an ADHD clinic in GP practices to review and manage children and adolescents with ADHD was also suggested as a way to engage GPs more in the management of ADHD including the consideration of drug holidays.

Findings indicated a possible connection between lack of uptake of practitioner-initiated drug holidays and lack of resources about ADHD drug holidays for patients or parents to use (physical opportunity) and to address parents’ concerns (social opportunity). Discussions, which happened within secondary care, took the shape of verbal communication that might reflect an individual practitioner’s attitudes and views about drug holidays. Decision-making models are becoming increasingly important in attempts to increase engagement of patients and their families as partners in their care (Légaré et al. 2008). A Cochrane review reported that decision aids could increase patient’s involvement and were more likely to lead to informed values-based decisions (Stacey et al. 2014). Another systematic review has shown that decision aids improve knowledge, reduce decisional conflict, and stimulate patients to be more active in decision making without increasing their anxiety (O’Connor et al. 1999). Specifically in ADHD, a study examined the effect of a shared decision-making intervention with parents of children newly diagnosed with ADHD and found that parents were better informed about treatment options without increasing visit duration (Brinkman et al. 2013).

The degree of shared decision making between families of children with ADHD and consultants varied across studies (Brinkman et al. 2011; Fiks et al. 2010). A study examined the views of parents and clinicians towards shared decision making and reported that both had favourable opinions (Fiks et al. 2010). Parents described the process as a partnership between equals, with physicians providing medical expertise, and the family contributing in-depth knowledge of the child. In contrast, clinicians understood shared decision making as a means to encourage families to accept clinicians’ preferred treatment (Fiks et al. 2011). Therefore, it is important to give families of children with ADHD full balanced information about the advantages and disadvantages of considering planned drug holidays to make their own decisions. Therefore, designing a decision aid about ADHD drug holidays could be a useful tool to help both practitioners and families to have a productive discussion, avoid practitioners’ bias, address parents’ anxiety towards stopping the medication and allow informed decisions to be made in relation to drug holidays in ADHD.

### Practical recommendations for successful drug holidays

Practitioners who took part in this study talked about the timing, length and most suitable cases for drug holidays to test the continued need for medications as demonstrated in the following recommendations:

1. *Timing* The best time to interrupt the medication is over the school holidays (often over the summer holidays). Stressful times such as Christmas, when the family is distressed for any reason, and the beginning of any school year, especially at the start of junior/senior high school should be avoided. However, parents should be given the choice on whether they prefer to involve the school in assessing how the child is without medication at school.
2. *Length* Drug holidays should be at least 1 week long and continue as long as child behaviour is manageable.
3. *Frequency* Drug holidays should be offered first after being on medication for 2 years and then at least on an annual basis.
4. *Best age* Independent of age, the indication for medication continuation should be assessed annually within a drug treatment-free period. However, drug holidays are more advisable among older children because they are able to use psychological interventions during the breaks, they can self-assess their abilities to cope without the medication, they may not need medication anymore due to the development of executive functions.
5. *Decision making* The decision on whether or not to take a drug holiday should be a shared decision that empowers and takes into account parents’ and patients’ choices and involves them fully.
6. *Sudden or abruptly stopping* Stimulant medication such as methylphenidate can be stopped abruptly without the need to gradually decrease the dose.
7. *Follow-up and monitoring* The assessment of the impact of drug holidays on children’s behaviour and abilities to concentrate should involve feedback from parents and their children as well as close follow-up by practitioners who may choose to use an objective measure to assess the usefulness of drug holidays.
8. *Most successful cases*
  Drug holidays are more successful among stable cases where the child is symptom-free for a reasonable period of time.
  Drug holidays should be offered to everyone regardless of their type of diagnosis (i.e. hyperactive/impulsive type, inattention type, and combined type).
  Drug holidays are more successful when there is an agreement between the child and their parents about having a trial without the medication and where the surrounding environments (such as family members and school teachers) are supportive.
  Drug holidays might be more successful among children who are on a low dose of medication or those who don’t have other comorbidities (such as autistic syndrome or obsessive–compulsive disorder).

## Strengths and limitations

This was the first study that examined in a qualitative and systematic way the barriers, from practitioners’ point of view, for the discussion of formal drug holidays from ADHD medication in children and adolescents. Bearing in mind the potential benefits of taking breaks from ADHD medication on managing side effects and testing continuing need, we discussed a number of interventions that could possibly increase at least discussions about planned drug holidays if not undertaking them. However, the experience of drug holidays from ADHD medication was examined among practitioners based in one English County. Although similar shared-care arrangements are developed in other UK counties, the findings might not fully represent the perceptions and experiences of other practitioners inside or outside the UK. This study was not designed for the purpose of identifying a protocol or to give recommendations for how to implement a ADHD drug holidays. More research is needed specifically to draw a consensus among health professionals about how to implement successful drug holidays to test the continued need for medication.

## Future research

Future research should focus on designing education and training interventions and examining how they could be operationalised in primary care to increase GPs’ involvement in shared-care of children with ADHD and enforce their capabilities to discuss and plan drug holidays. Moreover, future research should focus on designing decision aid(s) about drug holidays that could provide practitioners with the opportunity to discuss planned drug holidays with families and allow parents and their children to make informed decisions on whether or not to try drug holidays.

## Conclusions

Using an evidence-based methodology, possible barriers to the discussion and uptake of formal drug holidays as recommended by guidelines for the purpose of assessing the continued need for ADHD medication were identified and opportunities do exist for improving engagement. A number of interventions could be used including training and educating GPs about ADHD and employing a decision-making tool that could help families make an informed decision on whether or not to consider drug holidays. The implementation of decision aids about ADHD drug holidays could have cost-effective implications for the NHS by helping to save resources via stopping the prescribing of unnecessary medication. The findings presented here have the potential to aid practitioners and policy makers to routinely recommend and support planned breaks from ADHD medication during the school holidays to test continuing need for medication.

## Electronic supplementary material

Below is the link to the electronic supplementary material.

Supplementary material 1 (DOCX 13 kb)

## Abbreviations

ADHD: Attention deficit hyperactivity disorder
CAMHS: Child and Adolescent Mental Health Service
GP: General practitioners
NICE: National Institute for Health and Care Excellence
AACAP: Academy of Child and Adolescent Psychiatry
ICSI: Institute of Clinical Systems Improvement
CQUIN: Commissioning for Quality and Innovation
