# Supplementary material for: What stops practitioners discussing medication breaks in children and adolescents with ADHD? Identifying barriers through theory-driven qualitative research
Source: Atten Defic Hyperact Disord. 2018 Jul 7;10(4):273–83. doi: 10.1007/s12402-018-0258-9 (PMC6223995; doi:10.1007/s12402-018-0258-9)
Supplement: Supplementary file 1 — Supplementary material 1 (DOCX 13 kb) [file 12402_2018_258_MOESM1_ESM.docx]

**Clinicians’ interview schedule**

**I would like to ask you a couple of questions to get us started**

1. Can you tell me a little about your involvement with ADHD patients?

2. What symptoms and others features do you normally see in patients with ADHD when they are diagnosed with the condition? At what age are patients first diagnosed with the condition?

**Next, I’d like to ask specifically about medication for ADHD**

Presumably you prescribe medication (methylphenidate or stimulant medication) for ADHD.

1. What improvements do you see after medication is first prescribed? How long does it take for these bene-fits/effects to show?

2. Is medication always the solution?

3. Do you have any concerns regarding the prescribing of medication for ADHD? If yes, what are these concerns (side-effects, long-term efficacy, drug use, drug tolerability)?

4. On balance, do the benefits of medication for ADHD outweigh any concerns or not? Why is that?

**Now, I’d like to seek your views about the Berkshire methylphenidate shared-care guidelines**

According to this guideline, GPs and consultants should review the treatment with ADHD patient and his/her parents at least annually

1. How often can these reviews practically take place?

**2.** Can you talk me through what normally happens or should happen in such a review?

**Now, I’d like to ask you more specifically about treatment withdrawal**

The Berkshire guidelines states that at each review the GPs or consultants should have discussions with the child and his/her parents to decide whether the medication is still needed or not and to consider withdrawal of medication taking into account the patient/parent choice.

1. One of the things I’d like to establish is what clinicians understand by “withdrawal of treatment”. What do you think is intended by the term “treatment withdrawal”? (Drug holiday, treatment discontinuation)? For how long it should last?

2. Have you ever undertaken reviews for treatment withdrawal?

3. Do you think it’s practical to consider “treatment withdrawal” for some ADHD patients?

If yes,

1. What are the reasons that lead you to consider periods off the medication (either as a drug holiday or stopping altogether)?

2. Do you think everyone is suitable for a trial off medication?

3. Does the type of ADHD impact on encouraging treatment withdrawal? How? In what way – could you tell me more? What are the other factors that are important?

4. Do patients or parents ever make the decision to try a period off treatment withdrawal?

5. Who decides *when* to actually stop – i.e. in terms of timing? What factors affect choosing a time to try a period off the medication? Are there any specific periods during the year that would make treatment withdrawal more effective/ is there a particular age at which treatment withdrawal (or a period of treatment) is more suitable?

6. Once agreed, what sort of information would you give to patients and their families regarding treatment withdrawal?

7. Do you usually follow up the patients during these periods? And how would you follow them up and how often?

8. If you do suggest a trial off medication, what do you do if parents of ADHD children disagree with you and want their children to stay on medication? How are the issues resolved? And what are the reasons they might give for wanting to continue giving medication to their children?

If not,

1. What do you think of the recommendation to try a period without treatment?

2. Do parents of children with ADHD ever ask you to consider a period off medication or stopping medication altogether?

3. What reasons do they give?

4. What happens to resolve any issues that might arise?

**I wondered if we could now focus on actual or potential outcomes of treatment withdrawal**

1. In your opinion and experience what are the outcomes of trying periods off methylphenidate among children and adolescents with ADHD (on symptoms and side-effects)?

2. How would you assess these outcomes?

**Finally, thinking about the guidelines for treatment withdrawal**

1. If you think that treatment withdrawal is a feasible option in the management of children and adolescents with ADHD, what are your recommendations for implementing and managing treatment withdrawal? (over and above what is outlined in the Berkshire shared-care arrangement)
